# Spontaneous Cerebrospinal Fluid Rhinorrhea: A Case Report

**DOI:** 10.7759/cureus.9401

**Published:** 2020-07-26

**Authors:** Hemanth Krishna Boppana, Tonya Welch, Chrystal Calderon

**Affiliations:** 1 Internal Medicine, Port of Spain General Hospital, Port of Spain, TTO; 2 Radiology, Eric Williams Medical Sciences Complex, Champs Fleurs, TTO; 3 Neurosurgery, Port of Spain General Hospital, Port of Spain, TTO

**Keywords:** cerebrospinal fluid rhinorrhea, spontaneous cerebrospinal fluid rhinorrhea, endoscopy, trans-sphenoidal repair

## Abstract

Cerebrospinal fluid (CSF) rhinorrhea refers to the loss of CSF through the nasal cavity. Its causes can be classified as either spontaneous or non-spontaneous. Spontaneous causes of CSF rhinorrhea include congenital anatomical defects and are extremely rare, accounting for less than 4% of reported cases. Following failure of conservative management, definitive treatment most commonly involves an endoscopic transsphenoidal repair of the defect. We present a case of spontaneous CSF rhinorrhea in a previously well 52-year-old female, which required surgical intervention due to failure of conservative management.

## Introduction

The causes of cerebrospinal fluid (CSF) rhinorrhea can be classified as either spontaneous or non-spontaneous. Spontaneous or non-traumatic causes include congenital anatomical defects related to the temporal bone, skull base, or dura mater [1]. Non-spontaneous or traumatic causes of CSF rhinorrhea are related to surgical and accidental trauma, tumors, or exposure to radiation therapy involving the base of the skull [1]. Early diagnosis and effective management are needed to prevent the life-threatening complications of CSF rhinorrhea, including bacterial meningitis and brain abscesses. We present a case of spontaneous CSF rhinorrhea in a previously healthy 52-year-old obese female.

## Case presentation

A 52-year-old female presented with a six-month history of progressive rhinorrhea from the left nostril. There was a six-month history of headaches, which aggravated by bending over, but never with any associated fever, neck stiffness, photophobia, or trauma. The remainder of the patient’s history was insignificant for any known medical co-morbidities, except an elevated body mass index (BMI) of 34.5. On examination, clear fluid poured from the left nostril when the patient moved from the supine to prone position. On fundoscopy, we noted a normal cup-to-disc ratio with the absence of papilledema. We were unable to access a beta-2 transferrin assay study to confirm the identity of the fluid, but biochemical analysis of the draining fluid showed that the glucose concentration of the fluid compared with that of blood glucose favored CSF.

**Figure 1:**
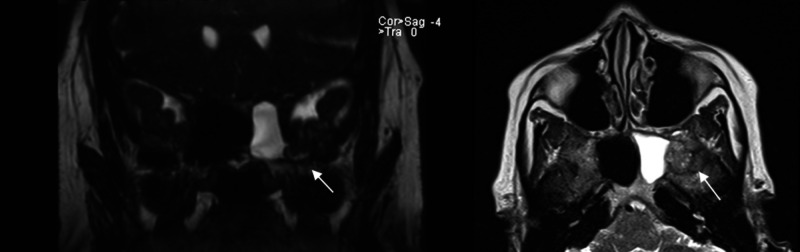
MRI of the brain demonstrating a small transsphenoidal temporal lobe encephalocele through the left lateral craniopharyngeal canal with associated drainage of CSF into the left side of the sphenoid sinus. CSF, cerebrospinal fluid

Computed tomography (CT) scan of the head at this time showed the presence of an empty sella with no evidence of skull fractures or obvious bone defects, but magnetic resonance imaging (MRI) with gadolinium enhancement revealed a CSF leak through the sphenoid bone between the left greater wing and the body (Figure 1), with no other abnormalities detected. An abnormal defect between the inferior aspect of the left temporal lobe to the left sphenoid sinus was noted along with a mild protrusion of the left temporal lobe into the left sphenoid sinus through this defect. CSF flowed through this defect to fill the left sphenoid sinus and further drain through the sphenoethmoidal recess. Imaging was consistent with a left lateral craniopharyngeal canal of Sternberg and an intra-sphenoidal encephalocele. Conservative treatment with the use of acetazolamide was initiated with no signs of improvement after two to three weeks. As a result, a surgical approach was utilized using a microscopic transnasal transsphenoidal approach under general anesthesia. Autologous graft from the lateral aspect of the thigh was utilized and laid along the left side of the cavity over the opticocarotid triangle with the placement of a lumboperitoneal shunt following the procedure. The post-operative period was uneventful with no evidence of CSF rhinorrhea, and the patient was subsequently discharged.

The patient returned one week later with bilateral CSF rhinorrhea accompanied by bouts of coughing. Open surgery with a left coronal incision was performed under general anesthesia, and protrusion of the medial temporal lobe was noted in keeping with the temporal lobe encephalocele depicted on imaging scans, and the harvested fascia lata graft was employed to repair the defect in the sphenoid bone. The temporal lobe was then allowed to fall over the fascia lata to ensure that its position was retained. Before securing the bone flap and closure of skin, duraplasty was employed to create a watertight closure and the lumbar drain was discontinued. Post-operation regime included the use of broad-spectrum antibiotics, acetazolamide, and strict bedrest with head elevation. The post-operative period was uneventful, and the patient displayed no evidence of recurrent CSF leak on further follow-up.

## Discussion

CSF rhinorrhea occurs due to an abnormal communication between the subarachnoid space and a defect in the skull base, leading to the loss of CSF through the nasal cavity [1]. Spontaneous or non-traumatic CSF rhinorrhea is extremely rare and accounts for only 4% of all reported cases of CSF rhinorrhea [2]. Spontaneous CSF rhinorrhea has been associated with elevated BMI and intracranial hypertension (ICH) [3,4]. The pathogenesis of CSF rhinorrhea is unclear, but previous studies have hypothesized that prolonged ICH may lead to defects in the skull base over time. These defects coupled with ICH can cause herniation of the dura mater into the bony defects, weakening the dura mater and making it more prone to dural tears and thus leading to a dural-mucosal fistula [5]. Similarly, obesity causes increased intra-abdominal pressure, leading to the elevation of the diaphragm and thereby leading to increased pleural and cardiac pressures, thus decreasing venous return from the brain to the heart and causing ICH [3,6]. The patient in the present case had an elevated BMI and complained of headaches that were worse on bending over, pointing to possible ICH, although the patient had no signs of ICH on examination.

The gold standard for detecting the presence of CSF is by testing for beta-2 transferrin or beta-2 trace protein [7,8]. Beta-2 transferrin is exclusively found in CSF, perilymphatic fluid, and the vitreous humor of the eye, with a reported sensitivity of 100% and a specificity of 95% [7]. In the case of our patient, we were unable to assess beta-2 transferrin testing and resorted to using comparative blood glucose concentrations of the draining fluid to the blood. The presence of glucose in secretions indicates the presence of CSF. However, detecting the presence of glucose in secretions is not recommended as a confirmatory test due to low diagnostic specificity and sensitivity and due to false-negative results in the case of bacterial contamination or false-positive results in diabetic patients [7]. Therefore, detection of glucose in CSF rhinorrhea cannot be used on its own to diagnose a CSF leak and requires concurrent clinical and radiographic evidence [9].

Radiological diagnostic techniques include the use of high-resolution CT and MRI scans. High-resolution CT and MRI scans are the most reliable means of differentiation between spontaneous and nonspontaneous CSF rhinorrhea. CT and MRI can assist in localization of leaks, especially when associated with fractures of surrounding bone or tumors, but do not demonstrate the leakage itself [2,7]. CT/MR cisternography is the gold standard for the detection of CSF leaks as it can identify the size, location, and quantity of the leak but is an invasive procedure and thus considered unnecessary if the diagnosis is supported by both the clinical presentation and imaging findings on CT and MRI [7,10]. In the case of our patient, CT scan was found to be non-diagnostic. MRI with gadolinium enhancement was required to identify the leak and the diagnosis of CSF rhinorrhea was confirmed through MRI findings and the presence of glucose in the CSF.

Treatment of CSF rhinorrhea usually involves an initial conservative approach, which, if failed, is followed by a surgical approach. Conservative treatment of CSF leaks consists of the use of acetazolamide along with prolonged bed rest with elevation of the head, which can decrease intracranial pressure [11]. Surgical intervention can involve either an endoscopic/extracranial approach or an intracranial approach. Intracranial approach carries increased morbidity and failure rates of 20-40%, whereas an endoscopic approach has less associated morbidity and a success rate of 90-100% [12-14]. A study that looked at 193 cases of treated CSF leaks over a period of 21 years concluded that the overall success rate of endoscopic repair was 98%, which, combined with an associated low morbidity, reinforced endoscopic repair as the standard of care for repair of CSF leaks [15]. However, it should be noted that an intracranial approach comes with its own advantages, including wide visualization of the leak site, and allows for direct repair of the leak. The current recommendations suggest initial treatment of CSF leaks using endoscopic repair, with extracranial repair reserved if indicated or following failure of endoscopic repair [16]. Adjunctive techniques to surgery that are commonly used to decrease morbidity and increase success rates include the use of antibiotics, diuretics, lumbar drains, and prolonged bed rest with elevation of the head [11,17]. In the present case, we initially performed an endoscopic repair using a transnasal transsphenoidal approach with the placement of an autologous graft over the opticocarotid triangle. This was followed by an open surgical approach when the patient represented a week later with bilateral CSF rhinorrhea, where the defect in the sphenoid bone was repaired using a fascia lata graft. We were unable to identify the cause of the recurrence of the CSF leak following the initial endoscopic repair.

Early diagnosis and prompt treatment of CSF rhinorrhea are important to prevent complications such as meningitis, intracranial sepsis, and abscesses, which are associated with high mortality rates. The overall rate of ascending meningitis associated with CSF leaks is 19% [18]. Currently, there is no evidence for the use of prophylactic antibiotics in the prevention of meningitis [19]. With successful surgical repair of CSF leak and an uneventful postoperative period, patients usually have a favorable prognosis. It is important to note that patients with CSF leaks are required to have 13-valent pneumococcal conjugate vaccine (PCV-13) and 23-valent pneumococcal polysaccharide vaccine (PPSV-23) eights months apart especially in the case of cranial CSF leaks due to the presence of a communication between the brain and surrounding structures and the oropharynx and nasopharynx [20].

## Conclusions

Spontaneous CSF rhinorrhea is a rare condition usually associated with an elevated BMI and ICH. The gold standard for the detection of CSF in secretions is beta-2 transferrin testing; however, CT/MR cisternography is the imaging test of choice as it can accurately identify the CSF leak. Prompt diagnosis and treatment of CSF leaks are necessary to prevent complications including ascending meningitis, which is associated with high mortality rates.
